# Development and Validation of an Argon Triple Point Apparatus with a Novel Automatic Pressure Control System

**DOI:** 10.3390/s25051411

**Published:** 2025-02-26

**Authors:** Ivan Matas, Lovorka Grgec Bermanec, Danijel Šestan, Jovan Bojkovski, Vincencij Žužek

**Affiliations:** 1 Laboratory for Process Measurement, Faculty of Mechanical Engineering and Naval Architecture, University of Zagreb, 10000 Zagreb, Croatia; lovorka.grgec@fsb.unizg.hr (L.G.B.); danijel.sestan@fsb.unizg.hr (D.Š.); 2 Laboratory of Metrology and Quality Ljubljana, Faculty of Electrical Engineering, University of Ljubljana, 1000 Ljubljana, Slovenia; jovan.bojkovski@fe.uni-lj.si (J.B.); vincencij.zuzek@fe.uni-lj.si (V.Ž.)

**Keywords:** ITS-90, realization, fixed point, triple point, argon, pressure, control, cryostat, comparison

## Abstract

This paper describes the development and validation of an apparatus for the realization of the triple point of argon (83.8058 K), with a novel automatic pressure control system for the liquid nitrogen cryostat. The automatic pressure controller, together with custom-made software, was developed and tested in the Laboratory for Process Measurement at the Faculty of Mechanical Engineering and Naval Architecture, University of Zagreb (FSB-LPM). Performance testing and characterization of the automatic pressure controller confirmed its suitability for precise and reliable control of gauge pressure in the cryostat. The characteristics and uncertainty of the measurement setup for the realization of the triple point of argon were validated through a bilateral hybrid comparison with the Laboratory of Metrology and Quality at the Faculty of Electrical Engineering, University of Ljubljana (MIRS/UL-FE/LMK). A long-stem quartz-sheathed standard platinum resistance thermometer was used as a transfer standard. The realizations of the International Temperature Scale (ITS-90) were compared in the subrange from the triple point of argon to the triple point of water. The comparison results show that resistance ratio (*W*) values determined by FSB-LPM at the fixed points of argon and mercury deviate from the MIRS/UL-FE/LMK values, within the determined combined uncertainty of the comparison.

## 1. Introduction

The triple point of argon (83.8058 K) is one of the cryogenic thermometric fixed points used for dissemination of the International Temperature Scale (ITS-90) [1]. It is also the fixed point with the lowest temperature that is used for calibration of long-stem standard platinum resistance thermometers (SPRTs), which are commonly used in national metrology institutes (NMIs) and secondary calibration laboratories as well.

The argon fixed point was officially introduced in the International Temperature Scale of 1968, in its Amended Edition of 1975 [2]. Since then, many different triple point of argon (Ar TP) cells and cryostats have been developed [3,4,5,6]. One of the first designs of the argon triple point sealed cell and its respective cryostat was introduced by LNE-Cnam [7]. This apparatus for Ar TP realization has a long tradition of use in many NMIs, and it has proven to be robust and reliable for the realization of the triple point of argon at the highest metrological level. Also, its performance and achievable uncertainties of the realization have been verified through several international comparisons [8,9,10,11,12,13]. According to the results presented in the final reports of the two most recent international comparisons on a European [13] and global level [10], uncertainties of the realization of the triple point of argon are in the range from 0.54 mK to 2.57 mK, expressed at the confidence level of 95% (*k* = 2).

In this system, the argon triple point equilibrium state is achieved in a hermetically sealed cell that contains 1 dm^3^ of pure argon. The argon cell is cooled by liquid nitrogen in a specially designed cryostat, down to the evaporation temperature of liquid nitrogen at atmospheric pressure (~77 K). The plateau of the triple point is achieved through a heat flux method, where no heating power is required. After the solidification of argon in the cell, the pressure in the cryostat is increased to increase the evaporation temperature of liquid nitrogen to a value just above the triple point of argon temperature. The achievement and duration of the triple point state of argon depend on the stability of the thermal conditions in the cryostat. Since liquid nitrogen in the cryostat constantly evaporates, regulation of the liquid nitrogen vapor pressure in the cryostatic vessel directly affects the temperature of evaporation.

The original version of the apparatus is equipped with a manual pressure regulator on the cryostat, consisting of a ball valve and a corresponding set of weights for the control of the outflow of the liquid nitrogen vapors. The manual pressure regulator operates based on the principle that a mass of weights creates a downward force on a metal ball that is placed on a conical outlet. If the force exerted by the weights exceeds the upward force, due to the pressure in the cryostat, acting on the metal ball, the nitrogen vapors are unable to exit the cryostat, leading to a rise in the gauge pressure. With correct placement of weights on the metal ball, the vapor pressure, and therefore temperature, of nitrogen in the cryostat can be controlled. This control system has been shown to be inflexible due to the limited number of weights, which cannot respond to small variations in atmospheric pressure, which have to be considered when aiming for the highest possible precision of temperature regulation. Several laboratories have already introduced alterations to the original cryostat design and pressure regulation method, in order to overcome the initial drawbacks of the apparatus [14,15,16,17].

**Figure 1:**
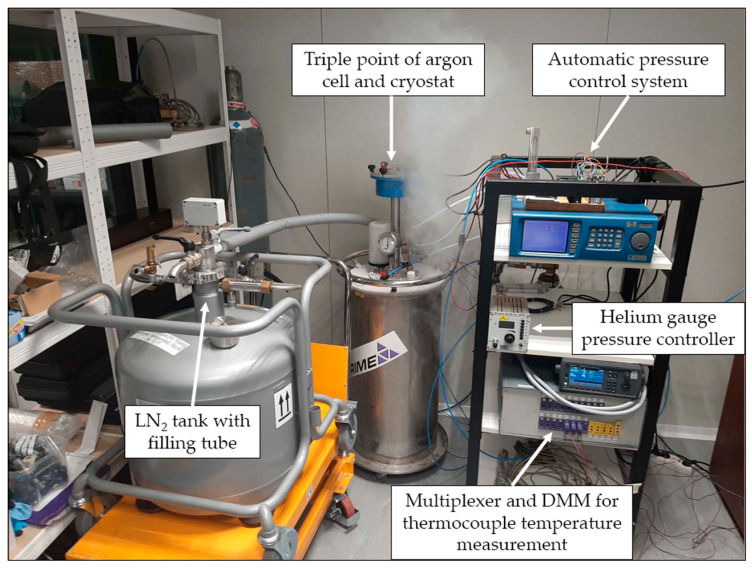
Argon fixed point measurement setup at FSB-LPM.

The above-described apparatus was acquired by the Laboratory for Process Measurement at the Faculty of Mechanical Engineering and Naval Architecture, University of Zagreb (FSB-LPM), which acts as a national calibration laboratory for temperature, humidity and pressure in Croatia [18]. With the goal of extending of the existing realization of ITS-90 in the laboratory to temperatures below the triple point of mercury (234.3156 K), FSB-LPM has developed a measurement setup for the realization of the triple point of argon, which can be seen in Figure 1.

This paper presents the design and verification procedure for the novel automatic pressure controller for the liquid nitrogen cryostat used for realization of the triple point of argon at FSB-LPM. The performance of the system and measurement uncertainties of the realization were evaluated through a bilateral comparison with the Laboratory of Metrology and Quality at the Faculty of Electrical Engineering, University of Ljubljana (MIRS/UL-FE/LMK), the national standard laboratory for the field of thermodynamic temperature and humidity. This hybrid comparison was also registered as EURAMET project no. 1681.

## 2. Materials and Methods

The original measurement setup for the realization of the triple point of argon at FSB-LPM was examined, and an automation of the existing pressure regulation was proposed as a needed improvement for easier and, therefore, more reliable operation of the system.

Several regulating principles have been examined during research at FSB-LPM, including a membrane pressure regulator, described in detail in [19], and a direct connection of the nitrogen vapor outlet to a pressure controller. Both initially proposed regulation principles were tested and disregarded due to the identified drawbacks. For the membrane pressure regulator, limiting factors for the regulation of sufficient precision were the characteristics of the membrane materials and the size of the pressure chamber.

When the outlet of the nitrogen vapors was directly connected to the pressure controller Druck DPI 515, the electromagnetic regulating valve did not allow sufficient outflow of vapors for reliable regulation of pressure in the cryostat. All of this stimulated the development of the new custom-made pressure controller at FSB-LPM. The developed pressure controller presents a low-cost, yet reliable and precise solution for the automation of the argon triple point realization process.

### 2.1. Measurement Setup at FSB-LPM

**Figure 2:**
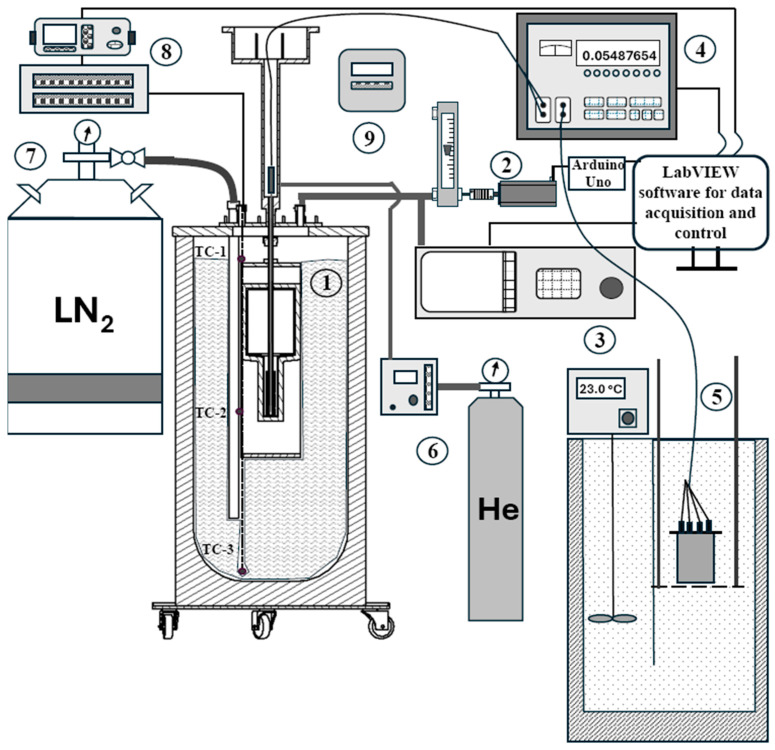
A schematic diagram of the measurement setup for the realization of the triple point of argon at FSB-LPM (components (1)–(9) are explained in the text above and below the Figure 2).

All components of the developed measurement system can be seen in the schematic diagram in Figure 2. The core components of the setup are the argon triple point cell and cryostat (manufacturer INM/Sorime, Ar-INM-36-FSB-LPM) (1). At the cryostat outlet, a novel pressure control system is installed that consists of a rotameter, Yokogawa RAGK41, whose needle valve is coupled to a stepper motor and controlled by an Arduino-based microcontroller (2). The gauge pressure inside the cryostat is measured at the same outlet using the precision pressure sensor Druck DPI 515 (3), which was calibrated against the gas pressure balance DHI PG7600 in the gauge pressure range 0–2 bar. The maximal deviation of the pressure transducer observed was −0.13 mbar at the upper limit of the working range (2000 mbar), with a maximal uncertainty of 0.11 mbar (*k* = 2). The observed change in the evaporation temperature due to the change in the gauge pressure of nitrogen vapors in the cryostat was approximately 5 mK/mbar. Thus, the contribution of the pressure transducer to the uncertainty of the achievement of desired thermal conditions in the cryostat is 0.6 mK (*k* = 2).

The thermometer inside the argon fixed point cell is connected to a precision thermometry bridge ASL F18 (4). The reference 100 Ω resistor Tinsley 5685A is kept at 23 °C in the thermostated oil bath Kambič OB-50 (5). To prevent air and water condensation in the thermometer well, it is filled with helium gas and kept at a gauge pressure of around 30 mbar. The gauge pressure of helium gas is controlled using the regulator Druck DPI 530 connected to a helium gas container (6). The pressure transducer of the regulator Druck DPI 530 was also calibrated with the gas pressure balance DHI PG7600 in the gauge pressure range of 0–2000 mbar. The maximal deviation of −0.18 mbar was observed at the upper limit of the working range, with a maximal uncertainty of 0.17 mbar (*k* = 2).

At the cryostat inlet, a liquid nitrogen container with a manual ball valve is connected via a transfer hose (7). The level of the liquid nitrogen inside the cryostat is monitored with three E-type thermocouples (TC1-3) accommodated inside a metal protective sheath, thermally anchored to the cryostat and connected to Keysight DAQ970A through a homemade multiplexer (8). Used E-type thermocouple wires of higher grade cover the range from −270 °C to 870 °C with an accuracy of ±1 °C. Thermocouple TC-1 is placed at the same level as the top of the fixed point cell, to monitor if the cell is fully immersed in liquid nitrogen. Thermocouple TC-2 is positioned at the level of the SPRT sensor in the thermometer well, to monitor the temperature of liquid nitrogen around the cell, and TC-3 is near the bottom of the cryostat, to monitor when liquid nitrogen is fully evaporated.

The atmospheric pressure during realization is measured with a Vaisala PTB220 absolute pressure sensor (9). The uncertainty of the measured atmospheric pressure is 0.06 mbar (*k* = 2), in the range from 950 mbar to 1100 mbar absolute. All measuring equipment are operated with the custom-made LabVIEW 2022 Q3 (version 22.3.1f8) software for continuous data acquisition and control.

For the realization of the triple point of mercury, an Isotech cell ITL-M-17724 is used, together with an alcohol-filled calibration bath, Isotech 915.

### 2.2. Novel Automatic Pressure Controller

**Figure 3:**
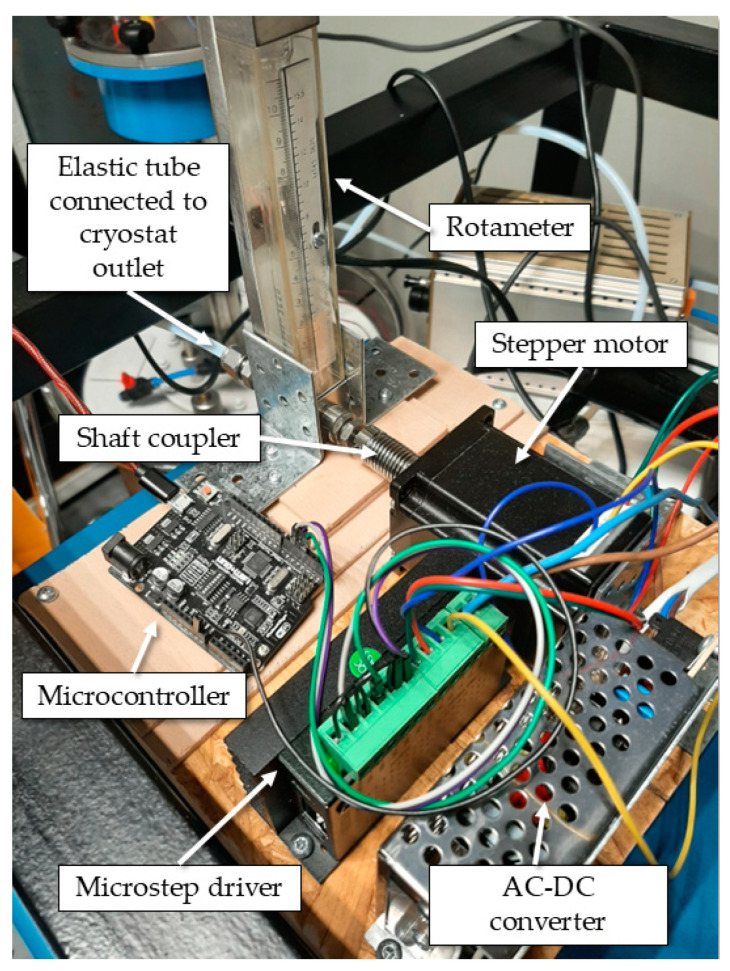
Novel pressure control system at FSB-LPM.

In order to automate pressure regulation of the nitrogen vapors in the cryostat, the manually operated valve with weights was replaced with a rotameter positioned at one of the cryostat outlets. The rotameter was chosen due to the high precision of its needle valve. To control the opening or closing of the rotameter valve automatically, the rotameter shaft was coupled with the shaft of the stepper motor. The novel pressure control system can be seen in Figure 3.

**Figure 4:**
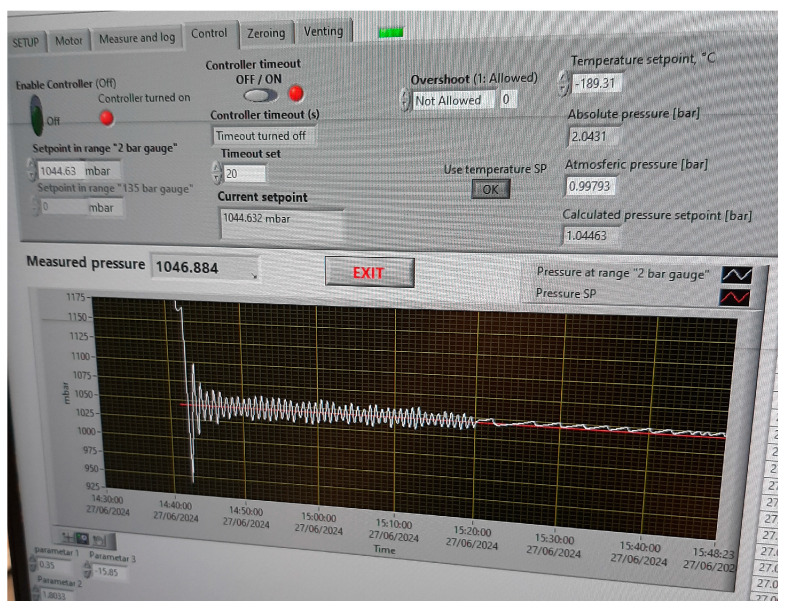
Pressure measurement and control software.

The pressure regulating principle of the novel controller is based on the regulation of the outflow of nitrogen vapors from the cryostat, depending on the nitrogen vapor pressure measured in the cryostat. An Arduino-based microcontroller controls the stepper motor through a microstep driver. LabVIEW 2022 Q3 (version 22.3.1f8) softwarewas programmed to acquire data from the pressure sensor Druck DPI515 and to send commands to the stepper motor. One step of the stepper motor turns the rotameter valve by approximately 0.1°, which enables precise regulation of the opening and closing of the valve. The maximal opening of the rotameter valve equals 51,200 steps of the stepper motor from the fully closed position. The front panel of the software can be seen in Figure 4.

From the difference between the setpoint and measured gauge pressure, the LabVIEW 2022 Q3 (version 22.3.1f8) softwarecalculates the required number of rotation steps by which the electric motor must rotate, and in which direction to close or open the rotameter valve, thus increasing or decreasing the pressure inside the cryostat. Based on the aforementioned pressure difference, the software also calculates the waiting time after each rotation, to take into account the dynamics of the pressure increase or decrease in the cryostat. The desired control precision can also be adjusted by setting a minimum difference between the desired and set pressure (permissible deviation from the pressure SP), below which the software will not perform pressure control. The software also allows the calculation of the pressure setpoint from the entered nitrogen evaporation temperature, based on the integrated “CoolProp” data [20]. From the measured atmospheric pressure and gauge pressure in the cryostat, the software calculates absolute pressure and searches in the integrated “CoolProp” database for the evaporation temperature that corresponds to the calculated pressure. The operating range of the novel pressure controller is from 0 mbar to 2000 mbar of gauge pressure. The upper limit of the operating range is constrained by the maximal operating pressure of the cryostat. To ensure that the pressure in the cryostat does not, at any point, exceed the maximal operating pressure recommended by the manufacturer (3 bar absolute), the cryostat is equipped with the two caps with small bores for the nitrogen inlet and outlet, which should inhibit over-pressurizing of the cryostat. Also, the following safety measures were incorporated into the design of the whole measurement setup to prevent over-pressurization of the cryostat in the event of the LabVIEW software crashing. A pressure relief valve was added to the cryostat outlet, and was set to a gauge pressure of 2 bar. Also, when the stepper motor is unplugged from the power source, its shaft can be manually rotated to open the rotameter valve.

The pressure controller had to be tested and characterized prior to its use for the regulation of the vapor pressure of the liquid nitrogen in the cryostat. It had to be adjusted to the previously observed dynamics of the nitrogen pressure variations (rate of pressure increase and drop) in the cryostat, during the realization of the argon fixed point temperature. Also, the limits of the rotameter valve had to be considered when programming the software, so that the fully closed or fully opened position of the valve could be detected in a timely manner, to avoid damage. Furthermore, the controller’s stability and stabilization time were tested at different pressures in increasing and decreasing series. The time between the change in the pressure setpoint in the LabVIEW software and the timepoint at which the amplitude of the oscillations of pressure dropped below 3 mbar was regarded as the stabilization time of the pressure controller. The stabilization time of the pressure controller was tested at different pressure setpoints (SPs) in the range from 700 mbar to 1100 mbar of gauge pressure, as this pressure range is relevant for the process of realization of the triple point of argon.

### 2.3. Measurement Setup at MIRS/UL-FE/LMK

**Figure 5:**
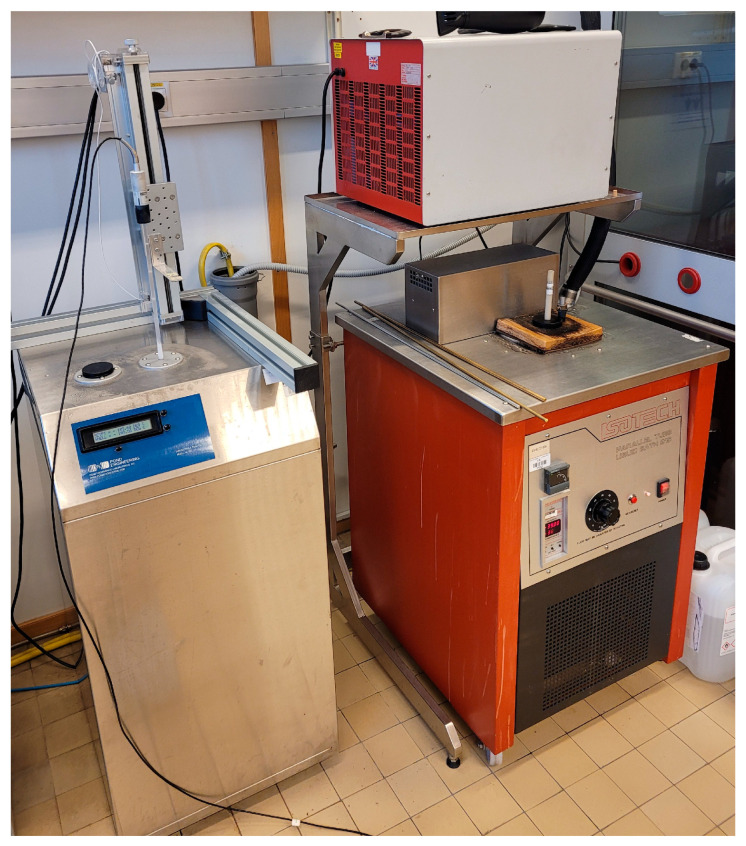
Triple point of argon cryostat (left) and triple point of mercury ethanol bath (right).

At LMK, a Pond Engineering K52 argon triple point cell and maintenance system are used. This is a completely automated system, cooled with liquid nitrogen (Figure 5). The triple point is achieved and maintained with an electrical heater that holds the cell temperature at −189.3 °C, ensuring a long-lasting plateau. The immersion depth of the thermowell is about 18 cm, and no contact gas, such as helium, is needed. The procedure used for the realization of the triple point is explained in the user manual.

The SPRT is measured with an ASL F900 AC resistance bridge. The bridge is paired with a reference resistor Tinsley type 5685A with a nominal resistance of 100 Ω, stored in an oil bath at (23 ± 0.002) °C. Acquisition and analysis of data is handled by a custom-made LabVIEW software package [21]. Additionally, for the measurements of the SPRT immersion profiles, a custom-made automatic vertical positioning system is used [22].

The mercury triple point is realized with a sealed Isotech mercury cell, which is contained in an Isotech ethanol bath (Figure 5). This setup is identical to the one used in LPM. The procedure for the realization of the triple point is as follows. The bath setpoint is first set to a few degrees below the freezing point, and the mercury is left to freeze overnight. Then, the setpoint is increased to a temperature of 0.3 °C above the melting point. When the mercury starts melting, as indicated by the monitoring SPRT, the SPRT is removed, and a room-temperature metal rod is inserted into the cell for one minute to achieve the “inner melt”. Then, the monitoring SPRT is reinserted in the cell, and measurements can commence once a stable melting plateau is reached.

### 2.4. Bilateral Comparison Protocol

The performance and measurement uncertainty of the entire measurement setup developed at FSB-LPM were verified through a bilateral hybrid comparison. The purpose of this hybrid comparison was to compare realizations of the ITS-90 in the triple point of argon (83.8058 K) and the triple point of mercury (234.3156 K), through the calibration of the SPRT at MIRS/UL-FE/LMK, acting as the Issuing NMI, and FSB-LPM, acting as the Applicant NMI.

According to the agreed technical protocol, the transfer standard (quartz-sheathed long-stem SPRT Hart Scientific 5681, serial number 1408) was provided by MIRS/UL-FE/LMK. The transfer standard was first calibrated at the Issuing NMI, then at the Applicant NMI, and again upon return to the Issuing NMI. The resistance ratio *W*(FP*_i_*) values determined at each participating laboratory were compared. Prior to measurements for each fixed point, the resistance of the transfer standard in the triple point of water had to be measured.

The calibration of the transfer standard proceeded from higher to lower temperatures. Both fixed point temperatures had to be realized at least three times to ensure the repeatability of the realizations. All SPRT resistance measurements in the fixed point cells were performed at a specific time after the beginning of the plateau, according to the recommendation in [9]. To assure that the conditions in the triple point cell during different realizations were as close as possible, measurements in the triple point of mercury had to be made in the period from 60% to 80% of the total duration of the plateau. In the case of the triple point of argon, measurements had to be made in the period from 20% to 40% of the total duration of the plateau. Also, the heat flux (immersion) profile had to be measured for the bottom-most 5 cm of each fixed point cell and compared to an ideal hydrostatic head correction.

## 3. Results and Discussion

**Figure 6:**
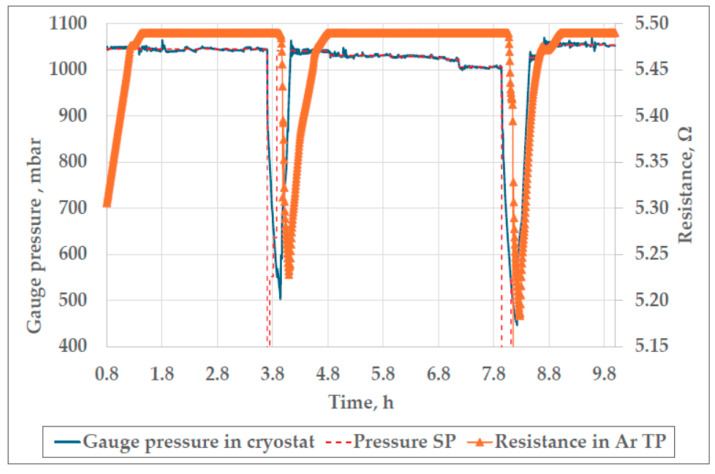
Testing of the pressure controller’s performance during the realization of the Ar TP.

**Figure 7:**
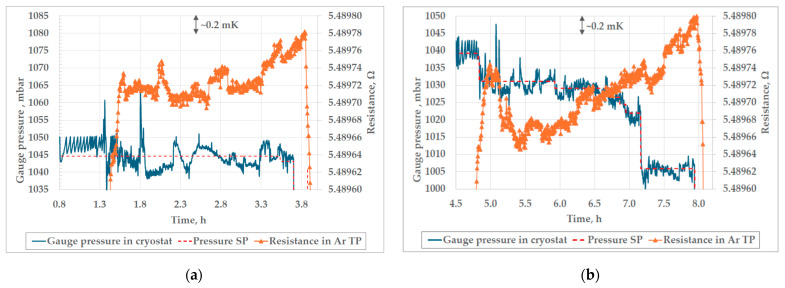
The controlled gauge pressure in the cryostat during the 1st realization (**a**) and 2nd trial realization (**b**) of the triple point of argon.

Upon completion of the design phase of the pressure controller development, it was tested to ensure that it was fit for its intended purpose. The results of the pressure controller tests are shown in Figures 6, 7 and 9. Also, the thermal conditions in the cryostat and the cell were examined (Figures 10 and 11) to fully characterize the Ar TP system. The overall performance of the whole measurement setup for the realization of the Ar TP was verified through a bilateral comparison, whose results can be seen in Table 2.

### 3.1. Testing and Characterization of Pressure Controller

Figure 6 shows the performance of the controller during the realization of the argon fixed point. As can be seen from Figure 6, with the use of the newly designed pressure control system, two plateaus of the triple point of argon were achieved with one batch (50 kg) of liquid nitrogen in the cryostat. Although the goal was to achieve three plateaus of Ar TP, the third plateau that can be seen at the right end of Figure 6 was interrupted due to the lack of liquid nitrogen in the cryostat, which caused an excessive temperature rise. The two plateaus of Ar TP, which were considered fit for further investigation, are shown in Figure 7.

Also, it can be seen from Figure 6 that the novel pressure controller is capable of efficient control after a sudden drop or increase in pressure, which is crucial for the realization of more than one Ar TP plateau during one filling of the cryostat. A delay of up to 9 min can be observed after the vapor pressure (and evaporation temperature) drops in the cryostat, until the temperature in the cryostat and argon cell starts to decrease. This delay can be attributed to the thermal inertia of the liquid nitrogen cryostat and the Ar TP cell, which depends on respective thermal conductivities, densities and heat capacities. The observed delay can also be attributed the latent heat of liquid argon, which must have dissipated from the cell for the solidification of argon and the start of the temperature decrease in the cell. After the first plateau of the triple point presented in Figure 6, the gauge pressure in the cryostat decreases by 550 mbar, which corresponds to the decrease in the evaporation temperature of liquid nitrogen of approximately 3 K. Since there is no forced circulation in the cryostat, the liquid nitrogen is stirred only by nucleate boiling, which limits heat exchange, and the temperature of about 50 kg of liquid nitrogen has to be decreased. Also, the Ar TP cell is thermally insulated with 1 cm of polyurethane foam to minimize the influence of thermal fluctuations in the cryostat on the argon inside the cell. This leads to a 9 min delay in the temperature drop in the Ar TP cell. After the second plateau, seen in the middle of Figure 6, the pressure drop is 550 mbar, but the cryostat has less liquid nitrogen due to evaporation, which speeds up the cooling process and shortens the delay to 4 min. To achieve as many plateaus of the triple point as possible with one batch of liquid nitrogen (50 kg), the pressure drop between the plateaus should be greater to ensure a lower temperature of liquid nitrogen and faster re-solidification of argon in the cell.

Figure 7 shows controlled gauge pressure during two trial realizations of the triple point of argon. The largest amplitude of the pressure oscillations observed during the first plateau in Figure 7a, with a total duration of 2.3 h, was 16.2 mbar. The average deviation of the measured pressure from the SP during the whole plateau was 7.5 mbar, with a standard deviation of 4.0 mbar. During the second plateau of the Ar TP, presented in Figure 7b, the largest amplitude of pressure oscillations was 5.7 mbar. The average deviation of measured pressure from the pressure SP was 3.2 mbar for the whole duration of the plateau (2.4 h), with a standard deviation of 1.4 mbar. The amplitude and duration of the oscillations of pressure in the cryostat are dependent on the pressure measuring interval and permissible deviation from the pressure SP, which can be adjusted in the software. During both plateaus presented in Figure 7, the measuring interval was set to 3 s and the permissible deviation to ±5 mbar. It was observed that, by setting a shorter measuring interval and a smaller permissible deviation, the amplitude and duration of the pressure oscillations were decreased. The lower limit of the measuring interval was 3 s, since further reduction led to overfilling of the instrument buffer and loss of communication between the instrument and software. The permissible deviation was not lowered below ±3 mbar, since more precise regulation was not needed, and further reduction could lead to destabilization of regulation.

Although a slightly longer duration of the second plateau could be attributed to the more stable pressure regulation, which is indicated by a smaller average deviation from the pressure SP, further investigations of the influence of the stability of pressure in the cryostat on the duration of the plateau of the Ar TP are needed. Also, during the duration of the second plateau, the gauge pressure SP was additionally decreased, which lowered the temperature in the cryostat as an attempt to extend the duration of the plateau. The second plateau was slightly extended, but a larger slope was observed, so an additional investigation is needed to determine the optimal pressure SP for achievement of the longest plateau.

The results of this test show that the presented laboratory-developed pressure control system is able to control the vapor pressure of liquid nitrogen in the cryostat in such a way that the plateau of the triple point of argon can be realized with satisfying duration and stability.

**Figure 8:**
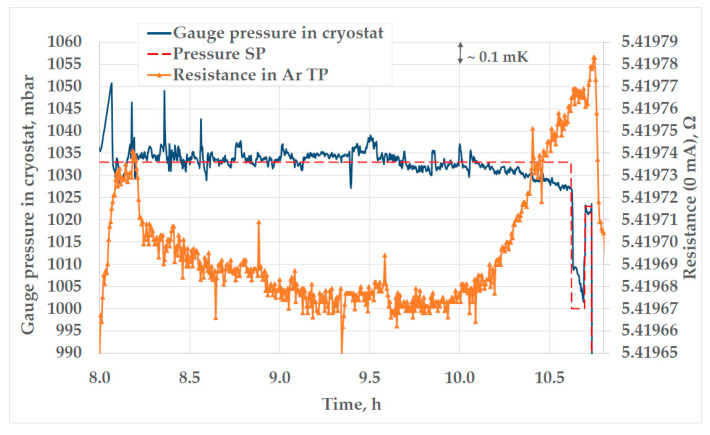
The longest plateau of the Ar TP achieved with the novel automatic pressure control system.

Currently, the longest plateau of the Ar TP that has been achieved with the novel automatic pressure regulator during a trial realization is 2.72 h, with a slope of 1.03 mK. The recording of this plateau is presented in Figure 8.

**Figure 9:**
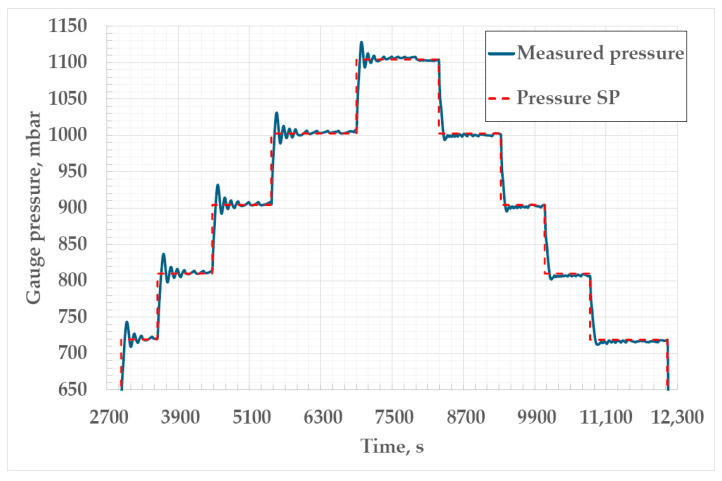
The stabilization time test of the pressure controller in the range of 700 mbar to 1100 mbar of gauge pressure.

The results of the stabilization time test of the pressure controller can be seen in Figure 9 and Table 1. The deviations of the measured pressure from the SP presented were observed in the period of at least 5 min after stabilization was achieved. The stabilization time of the controller was slightly higher when achieving an increased SP, and this can be seen in Figure 9 as larger deviations from the setpoint. Nevertheless, the stabilization time did not exceed 5 min, as can be seen in Table 1 This can be attributed to the fact that the increase in pressure in the cryostat after the closing of the valve on the nitrogen vapor outlet depends mostly on the nitrogen evaporation rate. When decreasing the pressure SP, the stabilization time of the controller was less than 2 min for all pressure points. After changing the pressure SP to lower values, the valve on the outlet was opened and nitrogen vapors exited the cryostat, creating an instant drop in gauge pressure, which contributed to faster stabilization at the new SP. Stable regulation of pressure at all pressure points was detected when oscillations of the pressure from the SP were lower than ±3 mbar. During the conduction of the test, the pressure measuring interval was set to 3 s and the permissible deviation to ±3 mbar. The results of this test, shown in Table 1, confirm that the developed pressure control system is capable of fast stabilization at any pressure SP within the range of 700 mbar to 1100 mbar of gauge pressure, which is relevant for the realization of the triple point of argon in the cryostat. The average deviations from the SP after stabilization are within the preset permissible deviation limits.

### 3.2. Thermal Conditions in Cryostat and Cell

**Figure 10:**
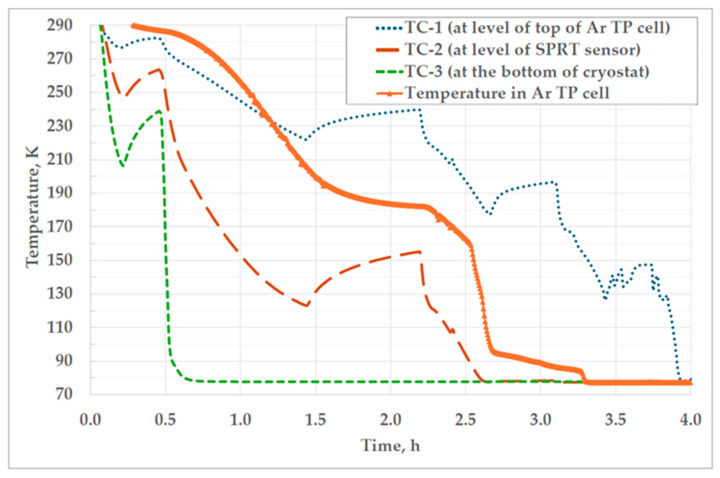
The temperatures of liquid nitrogen measured at three specific levels in the cryostat (TC-1–TC-3) and the temperature recorded by the thermometer located in the thermowell of the Ar TP cell, both observed during the cryostat filling process, which had to be interrupted twice for the additional pressurization of the LN_2_ tank.

**Figure 11:**
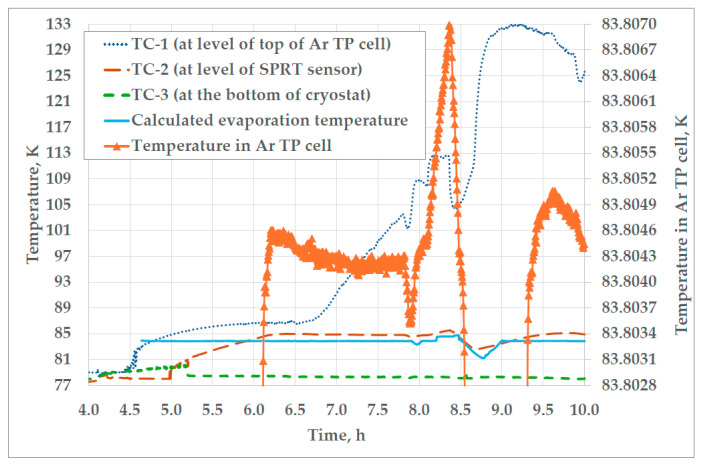
The temperatures in the cryostat and the cell during realization of the Ar TP (only the temperature in the Ar TP cell is shown on the right y-axis).

The thermal conditions inside the cryostat during filling are presented in Figure 10, and during realization of the Ar TP in Figure 11.

The cryostat filling process can take up to 4 h, depending on the rate of filling that is controlled by a manual valve on the liquid nitrogen container. In the beginning, the cryostat is filled slowly, to avoid over-pressurizing due to intensive evaporation of liquid nitrogen when in contact with the warm cryostat walls. As the cryostat becomes colder, the filling can be accelerated by further opening of the valve. The acquirable rate of filling through a transfer syphon also depends on the gauge pressure in the tank. During the filling process presented in Figure 10, the gauge pressure in the LN_2_ tank had to be increased twice due to the insufficient filling rate. Since the LN_2_ tank was not equipped with a pressure build-up regulator, the cryostat filling process had to be stopped to pressurize the LN_2_ tank with the nitrogen gas from the 200-bar bottle. During pressurization of the LN_2_ tank, the temperature in the cryostat increased, which can be seen in Figure 10 from the TC-1 and TC-2 values in the periods of 0.2–0.5 h and 1.4–2.2 h. The overall filling time was increased by approximately 1.1 h due to pressurization of the LN_2_ tank.

According to the filling procedure proposed by the manufacturer, the filling has to be stopped just once when the cryostat is filled to the level of the lower third of the Ar TP cell, which can be seen in Figure 10 when the temperature of the thermocouple TC-2 reaches the evaporation temperature of liquid nitrogen (~77 K at atmospheric pressure). The pause in the filling process has to last at least 30 min to ensure that the argon solidifies properly in the lower part of the cell, in the vicinity of the SPRT sensor. After the waiting time has elapsed, the filling process can be continued until the thermocouple TC-1, located at the level of the top of the Ar TP cell, reaches the temperature of ~77 K.

After the termination of the filling process, the gauge pressure in the cryostat was set to 1045 mbar. With an atmospheric pressure of 1000 mbar, the absolute pressure in the cryostat (2045 mbar) corresponds to an evaporation temperature of 189.3 °C (according to the “CoolProp” data for nitrogen), which is just slightly above the temperature of the triple point of argon. In those conditions, the temperature in the Ar TP cell slowly rises for 2 h until it enters the plateau of the triple point, which can be seen in Figure 11.

As can be seen from Figure 11, the Ar TP cell was not fully immersed in the liquid nitrogen during the whole realization, as the temperature of TC-1 (positioned at the level of the top of the cell) is higher than that of TC-2, which is positioned at the level of the lower third of the cell. This poses a temperature gradient alongside the Ar TP cell gradient that ranges from 2 K at the beginning of the plateau, to almost 30 K at the end. Despite this, the temperature uniformity and stability in the argon cell allow the achievement of a plateau with a duration of 1.63 h and a slope of 0.51 mK, which can be seen from the values of the temperature in the Ar TP cell, shown on the right y-axis of Figure 11. Although a longer plateau could be achieved with full immersion of the Ar TP cell, the achieved slope is comparable with slopes found in the literature [14,15]. This contributes to the cost-effectiveness of the presented setup, since the three plateaus of satisfying repeatability (0.154 mK at *k* = 1) can be achieved with approximately 75 kg of liquid nitrogen. In comparison to the trial realizations, where approximately 50 kg of liquid nitrogen was used and two plateaus of the Ar TP could be achieved, an additional mass of approx. 25 kg of LN_2_ had to be added to the cryostat in order to achieve the third plateau of the Ar TP during the comparison measurements.

Also, it can be observed that the temperature in the cryostat at the level of the SPRT sensor in the cell follows the changes in vapor pressure and, consequently, evaporation temperature. This validates the principle of the realization of the triple point of argon through the control of the vapor pressure of liquid nitrogen in the cryostat.

### 3.3. Results of Bilateral Comparison

A summary of the results of the bilateral comparison is shown in Table 2. The values for resistance (*R*) and the resistance ratio (*W*) are averaged from the results obtained in three independent realizations of each fixed point temperature. The measurement uncertainties (*U*) shown in Table 2 correspond to the calculated resistance ratio (*W*), and are presented at a level of confidence of 95%. The resistance measured at both fixed points was corrected for self-heating and hydrostatic head, and corresponds to a measuring current of 0 mA.

**Figure 12:**
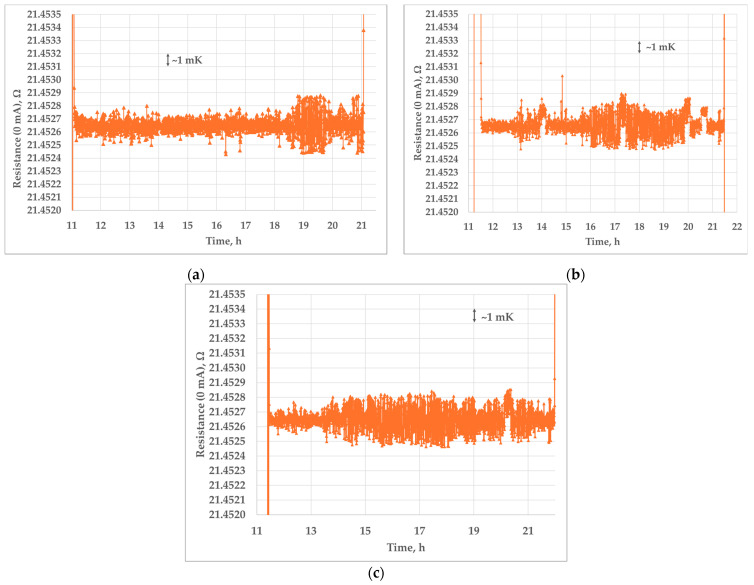
Plateaus of the triple point of mercury measured at FSB-LPM: 1st realization (**a**), 2nd realization (**b**) and 3rd realization (**c**).

Figure 12a–c show three independent realizations of the triple point of mercury at FSB-LPM. As can be seen from Figure 12, oscillations of the measured SPRT resistance are greater in the mercury cell, but they are still kept within the 3 mK range. The average temperature difference from the beginning to the end of all three 10 h plateaus of the triple point of mercury is 0.26 mK.

In all three realizations, relevant measurements of resistance were taken from the sixth to the eighth hour of the plateau.

**Figure 13:**
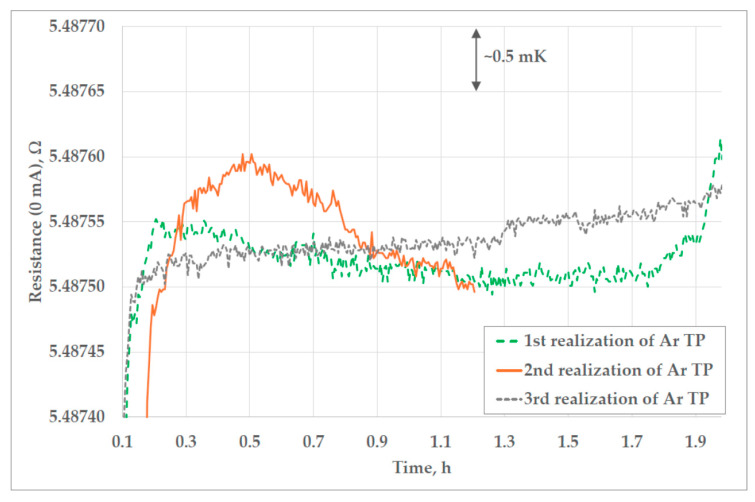
Plateaus of the triple point of argon realized at FSB-LPM.

Three realizations of the triple point of argon at FSB-LPM are presented in Figure 13. Relevant resistance measurements were conducted in the period from 40 to 80 min after the beginning of each plateau. The average duration of the first and the third plateau of the triple point of argon was around 2 h. The duration of the second plateau was shorter than usual, because three realizations of the triple point of argon had to be made with one batch of liquid nitrogen. The second plateau was aborted because of the conduction of the immersion profile test. The average temperature difference from the beginning to the end of all three plateaus was 0.36 mK.

**Figure 14:**
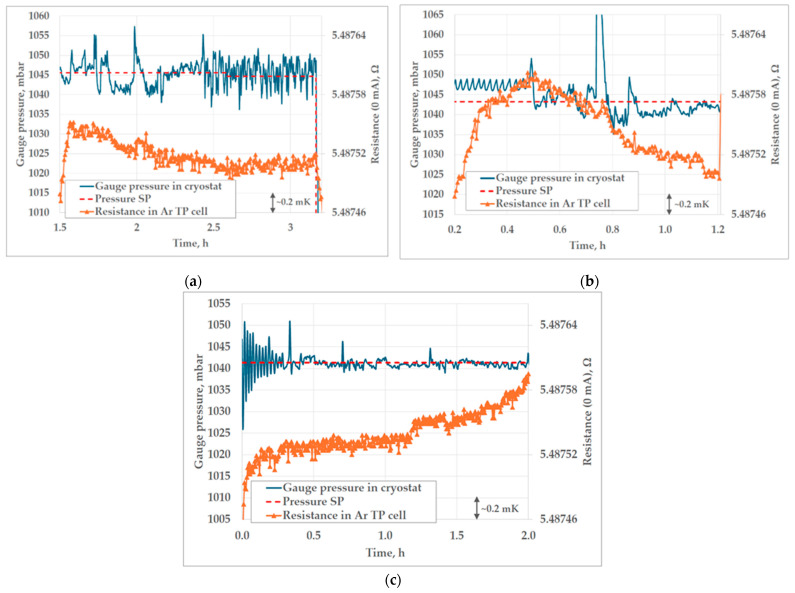
The gauge pressure in the cryostat during realizations of the triple point of argon at FSB-LPM: 1st realization (**a**), 2nd realization (**b**) and 3rd realization (**c**).

Figure 14 shows the gauge pressure in the cryostat during all three realizations of the triple point of argon at FSB-LPM. The standard deviation of the absolute difference between the measured gauge pressure and the pressure SP during all three realizations was lower than 5 mbar. The gauge pressure SP was adjusted for the duration of all three plateaus, to accommodate the oscillations of atmospheric pressure. The evaporation temperature of liquid nitrogen was kept constant, at 83.85 K, which corresponds to an absolute pressure of 2045.13 mbar, according to the “CoolProp” database.

**Figure 15:**
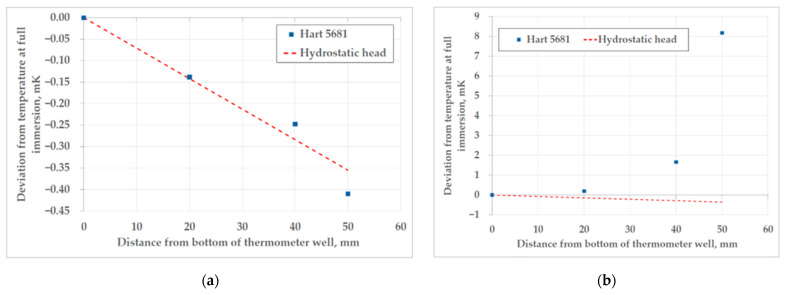
The immersion profiles for the fixed point of mercury (**a**) and argon (**b**) at FSB-LPM.

The results of the heat flux (immersion) profile tests, performed in both fixed point cells, can be seen in the Figure 15. The immersion profile of the triple point of mercury follows the ideal hydrostatic head correction, with no significant deviation.

During the immersion profile test in the Ar TP cell, the thermometer head protection tube, normally filled with helium, had to be opened, in order to change the position of the SPRT in the well. This caused significant disturbance of the thermal conditions in the cell, because the leaked-out helium from the thermometer well had to be replaced with helium from the bottle at room temperature. The results of the immersion profile test for the triple point of argon are presented in Figure 15b. Future investigation will include a repetition of the immersion profile test with a higher value of gauge pressure of helium in the thermometer well, to determine its influence on the immersion profile.

Table 3 presents the uncertainty budgets of both participating laboratories for the measurements in the triple point of mercury and triple point of argon. For the MIRS/UL-FE/LMK, for the triple point of argon and triple point of mercury, detailed budget of uncertainty analysis can be found in the final report of the EURAMET 1318 project, which is approved for equivalence [13]. FSB-LPM has also successfully participated in the above-mentioned comparison (the laboratory was named the HMI at the time of comparison), with the triple point of mercury as the lowest-temperature fixed point, which verifies the presented uncertainties of the realization of the triple point of mercury. In the case of FSB-LPM, the largest uncertainty contributing factor when realizing the triple point of mercury was the influence of the bridge characteristics, including the largest standard deviation of all measurements, which was slightly higher compared to the usual values (0.597 mK at *k* = 1). The large noise detected during measurements for the TP Hg could be attributed to the fact that the SPRT picks up noise from the compressor of the bath’s external chiller, since these large oscillations were not present during measurements for the triple point of argon. However, further investigation is needed for identification of the exact cause of the observed noise, which includes shielding of the SPRT during measurements.

Apart from standard deviation, the uncertainty contribution of the resistance bridge at FSB-LPM included the results of the non-linearity test (0.175 mK, *k* = 1), the effect of alternating current (0.113 mK, *k* = 1) and resolution (0.028 mK, *k* = 1) [23]. The contributions of all uncertainty sources were estimated according to the recommendations from the literature [9,24].

**Figure 16:**
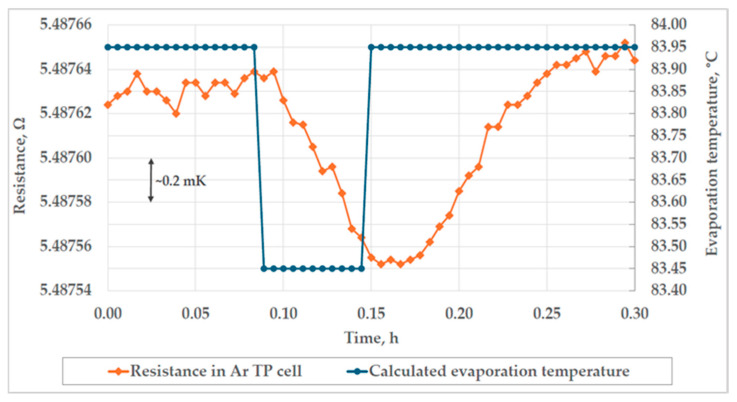
The results of the additional heat flux test.

In the case of the realization of the triple point of argon at FSB-LPM, the largest contribution to the uncertainty was from the influence of the heat flux test, which was found not to be appropriate for this type of Ar TP cell. The uncertainty contribution of the heat flux takes into account the difference between the ideal and the measured temperature for the bottom 20 mm of the thermometer well, and the influence of the change in pressure in the cryostat and, therefore, temperature around the triple point cell. The deviation from the ideal temperature profile contributed to the uncertainty of heat flux by 0.34 mK (*k* = 1). The disturbance of pressure in the cryostat of 100 mbar caused a change in the temperature of evaporation by 0.5 °C, as can be seen in Figure 16. The associated uncertainty, with assumed rectangular probability distribution, of the average temperature change in the Ar TP cell after the change in thermal conditions in the cryostat was 0.46 mK (*k* = 1). The results of the additional test for the calculation of the contribution of heat flux to the overall uncertainty of the realization are presented in Figure 16.

**Figure 17:**
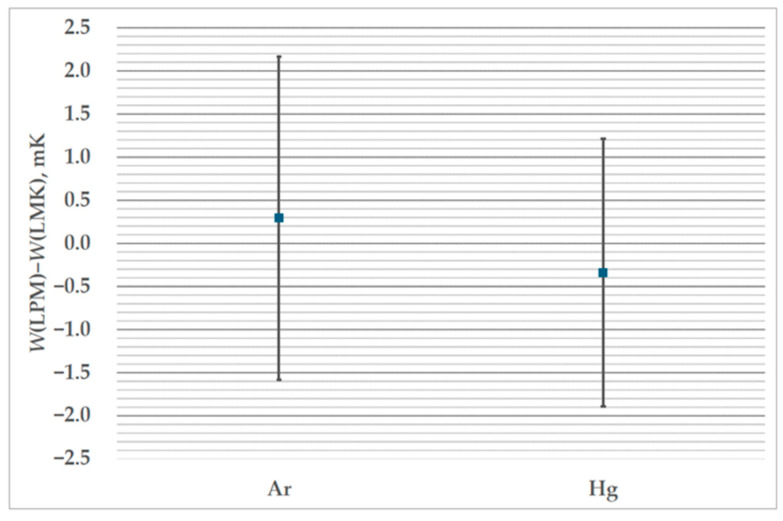
The results of the comparison: the deviation of *W*-values determined at FSB-LPM from the reference *W*-value determined from measurements at LMK.

Figure 17 presents the results of the comparison as deviation from the reference value, with error bars representing the combined uncertainty of both participating laboratories. The reference value has been determined by LMK, and its uncertainty includes the influence of the drift of the transfer standard. The drift was calculated from the difference in the *W*-values between the first and repeated measurements at LMK. The uncertainty of the drift is 0.049 mK (*k* = 2) at the triple point of mercury and 0.108 mK (*k* = 2) at the triple point of argon. Table 4 shows the resistance of the transfer standard measured at TPW before and after measurements at each participating laboratory. Values of the resistance *R* have been corrected for self-heating and hydrostatic head. The calculated uncertainty *U* is presented in terms of mK, and corresponds to the level of confidence of 95%. The uncertainties of the TPW values determined at MIRS/UL-FE/LMK were confirmed through their successful participation in the EURAMET.T-K7 comparison [25]. The results presented in Table 4 confirm that the transfer standard was stable during the course of the hybrid comparison.

The deviation of the value determined at FSB-LPM from the reference value for the triple point of argon is 0.29 mK, with a combined uncertainty of 1.87 mK. For the triple point of mercury, the deviation of the value determined at FSB-LPM is −0.34 mK, with a combined uncertainty of 1.55 mK. Both determined deviations are well within the combined uncertainty limits that include the uncertainty of the realizations from both participating laboratories (presented in Table 2) and uncertainty due to the drift of the transfer standard. The combined uncertainty was calculated as the square root of the sum of the squares of the three above-mentioned uncertainties.

In addition, in Figure 7b, Figure 8, Figure 11, Figure 13 and Figure 14a,b, peaks in measured resistance in the Ar TP cell are observable at the beginning of the plateaus. Further investigation is needed to identify the cause of these peaks and an efficient method for their mitigation. More precise pressure regulation, with the limitation of overshooting (observable in Figure 9), could possibly be achieved through the introduction of a PID control loop to the regulation software. Additionally, the realization procedure should be further investigated in order to decrease the above-mentioned peaks. The design of the cell and the absence of inner melt of argon in the cell make it more prone to the influence of external heat sources, especially at the beginning of the plateau. Therefore, the realization procedure should be adjusted accordingly. The stabilization of the cell at lower temperatures in the cryostat, prior to the realization of the Ar TP plateau, could lower the unwanted heat flux as the possible cause of the temperature peaks.

## 4. Conclusions

The development of the triple point of argon realization system at FSB-LPM has been presented. For the realization of the triple point of argon temperature in a hermetically sealed cell, a specially designed liquid nitrogen cryostat is used. The achievement and the stability of the plateau of the triple point of argon depend on the control of gauge pressure in the cryostat. The original cryostat is equipped with a manual pressure regulator, consisting of a set of weights, a metal ball and a conical outlet. The pressure regulation principle is based on controlling the outflow of nitrogen vapor from the cryostat. Due to the manual operation and limited number of weights, pressure regulation was found to be inflexible. Also, the cryostat was equipped with a Bourdon tube manometer with a resolution of 20 mbar for the measurement of pressure. All of this stimulated the development of a new pressure control system which keeps the original pressure regulating principle, but offers better flexibility, precision and complete automation of the pressure regulation.

The novel laboratory-developed pressure controller consists of a stepper motor, coupled to a rotameter connected to a nitrogen vapor outlet, and a precise pressure sensor. The regulation process is governed by custom-made software that acquires the gauge pressure in the cryostat from the sensor and controls the opening of the rotameter valve, and, therefore, the outflow of nitrogen vapor. The developed pressure control system was tested and characterized prior to its use for the realization of the triple point of argon. The results of the tests showed that the new pressure control system is able to achieve and control any pressure setpoint in the range from 0 mbar to 2000 mbar of gauge pressure in a timely manner, with oscillations from the setpoint lower than ±3 mbar. Thus, the flexibility of the pressure regulation is improved when compared to the manual pressure regulator, which had a sensitivity of ±12 mbar, when using the weight of the smallest mass.

The response of the novel pressure control system to sudden drops and increases in pressure show its suitability for the realization of the triple point of argon, since it is important to be able to realize more than one plateau within one filling of the cryostat. Through the automation of pressure regulation, complete control of the process of realization of the triple point of argon is enabled. The conventional cryostat was further improved with a temperature measuring system consisting of three thermocouples positioned at different levels, so the thermal conditions in the cryostat could be monitored during realization of the Ar TP.

To validate the performance and confirm the uncertainties of the argon triple point apparatus, a bilateral comparison with MIRS/UL-FE/LMK was organized and registered as EURAMET project no.1681. According to the agreed technical protocol of the comparison, the SPRT was calibrated at the triple point of argon and triple point of mercury, and the *W*-values of the participating laboratories were compared. The results of the comparison show that the *W*-values at the triple point of argon and mercury measured at FSB-LPM are within the combined comparison’s uncertainties. This validates the argon triple point apparatus with a novel pressure control system developed at FSB-LPM. Also, the performance of the mercury triple point cell and associated apparatus at FSB-LPM is confirmed. Through this research, FSB-LPM has expanded its measurement capabilities down to the temperature of the triple point of argon (83.8058 K).

Future investigations of the developed setup for the realization of the Ar TP will be focused on the determination of the optimal gauge pressure SP in the cryostat and optimal regulation parameters, with the possibility of the addition of a PID control loop to the regulation software, for the achievement of a plateau of the Ar TP with the smallest possible slope and the largest possible duration. Also, a re-evaluation of realization procedure is planned to better address the specifics of the design of the Ar TP cell. In the case of the mercury fixed point, future investigations will be focused on the determination of the cause of large oscillations during measurements. Both fixed points will be further investigated with the goal of decreasing the respective uncertainties of realizations.

## Figures and Tables

**Table 1:** The stabilization time and deviation from the SP of the pressure controller in the range from 700 mbar to 1100 mbar of gauge pressure.

Pressure SP	Stabilization Time	Average Deviation from Pressure SP After Stabilization
mbar	s	mbar
719.0	199	2.2
809.9	251	2.0
904.4	242	1.6
1002.5	235	1.8
1104.4	215	1.8
1002.5	99	1.8
904.4	103	2.5
809.9	99	3.0
719.0	102	2.1

**Table 2:** A summary of the measurement results of the bilateral comparison.

Participating Laboratory	*R*(Hg)	*W*(Hg)	Number of Realizations	*U* (*k* = 2)	*R*(Ar)	*W*(Ar)	Number of Realizations	*U* (*k* = 2)
	Ω	*-*		mK	Ω	*-*		mK
MIRS/UL-FE/LMK	21.452626	0.84415647	3	0.21	5.487475	0.21593103	3	0.26
FSB-LPM	21.452654	0.84415544	3	1.54	5.487543	0.21593311	3	1.85
MIRS/UL-FE/LMK	21.452678	0.84415716	3	0.21	5.487524	0.21593265	3	0.26

**Table 3:** The uncertainty budgets of both participating laboratories.

Uncertainty Source	Uncertainty Contribution (*k* = 1), mK			
	FSB-LPM	MIRS/UL-FE/LMK	FSB-LPM	MIRS/UL-FE/LMK
	Mercury		Argon	
Phase transition realization repeatability	0.012	0.041	0.154	0.038
Bridge (repeatability, non-linearity, AC quadrature)	0.633	0.027	0.230	0.027
Reference resistor stability	0.017	0.001	0.004	0.001
Chemical impurities	0.333	0.010	0.312	0.030
Hydrostatic head	0.082	0.035	0.038	0.016
Propagated TPW	0.225	0.060	0.058	0.020
SPRT self-heating	0.032	0.020	0.027	0.020
Heat flux	0.040	0.055	0.600	0.110
Insulation leakage	0.000	0.000	0.000	0.000
SPRT Pt oxidation	0.000	0.015	0.005	0.000
Gas pressure	0.000	0.000	0.000	0.000
**Combined uncertainty (*k* = 1)**	0.757	0.105	0.735	0.127
**Expanded uncertainty (*k* = 2)**	1.535	0.210	1.852	0.255

**Table 4:** A summary of the measurements of resistance in the TPW.

Participating Laboratory	*R*(TPW) on Receipt	*R*(TPW) After All Measurements in Fixed Points	*U* (*k* = 2)
	Ω	Ω	mK
MIRS/UL-FE/LMK	25.413144	25.413141	0.15
FSB-LPM	25.413157	25.413153	0.46
MIRS/UL-FE/LMK	25.413159	25.413151	0.15

## Data Availability

The datasets presented in this article are not readily available due to time limitations. Requests to access the datasets should be directed to the authors.

## Abbreviations

The following abbreviations are used in this manuscript:

FSB-LPM	Laboratory for Process Measurement, Faculty of Mechanical Engineering and Naval Architecture, University of Zagreb
MIRS/UL-FE/LMK	Laboratory of Metrology and Quality, Faculty of Electrical Engineering, University of Ljubljana
ITS-90	International Temperature Scale of 1990
SPRT	standard platinum resistance thermometer
Ar TP	triple point of argon
NMI	national metrology institute
LNE-Cnam	Laboratoire Commun de Métrologie
EURAMET	European Association of National Metrology Institutes
SP	setpoint
